# Trends in behavioral and biological risk factors for non-communicable diseases among adults in Bhutan: results from cross-sectional surveys in 2007, 2014, and 2019

**DOI:** 10.3389/fpubh.2023.1192183

**Published:** 2023-08-01

**Authors:** Supa Pengpid, Karl Peltzer

**Affiliations:** ^1^Department of Health Education and Behavioral Sciences, Faculty of Public Health, Mahidol University, Bangkok, Thailand; ^2^Department of Public Health, Sefako Makgatho Health Sciences University, Pretoria, South Africa; ^3^Department of Healthcare Administration, College of Medical and Health Science, Asia University, Taichung, Taiwan; ^4^Department of Psychology, University of the Free State, Bloemfontein, South Africa; ^5^Department of Psychology, College of Medical and Health Science, Asia University, Taichung, Taiwan

**Keywords:** trends, risk factors, non-communicable diseases, Bhutan, behavior

## Abstract

**Background:**

The study aimed to evaluate trends in the prevalence and correlates of risk factors for non-communicable diseases (NCDs; low physical activity, insufficient fruit/vegetable intake, current tobacco use, problem alcohol use, diabetes, hypertension, increased total cholesterol, and obesity) in Bhutan.

**Methods:**

Three repeat cross-sectional Bhutan STEPS surveys (*N* = 9,281) in 2007, 2014, and 2019 were analyzed.

**Results:**

The proportion of people with 3–8 NCD risk factors significantly decreased from 62.8% in 2007 to 32.6% in 2019 (*p* < 0.001), and the mean number of NCD risk factors significantly decreased from 3.0 in 2007 to 2.1 in 2019 (*p* < 0.001). In linear regression analyses by study year, older age (*p* < 0.001) was positively associated with eight NCD risk factors across all study years. Furthermore, male subjects were negatively (*p* < 0.01) and positively (*p* < 0.001) associated with eight NCD risk factors, respectively. Higher education levels (*p* < 0.05) were positively associated with eight NCD risk factors in 2007 and negatively associated with eight NCD risk factors in 2019 (*p* < 0.05). Employment (*p* < 0.001) and urban residence (*p* < 0.001) were positively associated with eight risk factors for NCD in 2019, while urban residence (*p* < 0.001) was negatively associated with eight NCD risk factors in 2014.

**Conclusion:**

The prevalence of eight NCD risk factors decreased in Bhutan over the past 13 years. Inadequate fruit and vegetable intake, problem alcohol use, and hypertension increased, current tobacco use, low physical activity, obesity, diabetes, and elevated total cholesterol decreased from 2007 to 2019. Several factors associated for eight and each individual NCD risk factor were identified, which can help guide interventions.

## Introduction

The majority (>85%) of deaths from non-communicable diseases (NCDs) occur in low-resource countries (1). In Bhutan, for example, 69% of all deaths in 2016 were caused by NCDs (2). Cardiovascular disease, cancer, respiratory disease, and diabetes contribute to more than 80% of all premature NCD deaths (1). Poor diet, tobacco use, harmful alcohol use, and low physical activity all increase the risk of dying from an NCD (1). In the Southeast Asia region, NCDs caused 7.9 million deaths in 2008 and are expected to increase by 21% over the next decade (3). Between 1990 and 2010, nearly all NCDs, in particular diabetes and coronary heart disease, increased at a higher rate in South Asia than globally (4). Considering the increase in NCDs in lower-resource countries in the East and Southeast Asian region, it is important to gain insight into the trends and local determinants of NCDs (3–6). In this context, national community-based trend data on NCD risk factors in Bhutan is needed.

A 2014 population-based study of adults in Bhutan (*N* = 2,822) found that 67% had inadequate fruit and vegetable intake (<5 servings/day), 6% did low physical activity, 7% were heavy alcohol users (>40 g for men/>20 g for women), 13% had elevated total cholesterol (≥190 mg/dL/or on anti-lipid medication), 36% had hypertension, 11% had elevated impaired fasting glucose, 33% were overweight/obese (≥25 kg/m^2^), and 24.8% were current tobacco users (7, 8). In Nepal in 2013, 27.7% of the population (15–69 years) had 3–8 NCD risk factors (98.9% inadequate fruit/vegetable intake, 25.7% elevated blood pressure, 22.6% elevated total cholesterol, 21.4% overweight or obesity, 18.5% current smoking, 3.6% elevated blood glucose, 3.4% low physical activity, and 2.0% harmful alcohol use) (9).

In a population (35–64 years) in the urban region of Delhi, India, the prevalence of overweight increased from 42.2% in 1991–1994 to 56.0% in 2010–2012, hypertension went from 23.0 to 42.2%, elevated fasting blood glucose went from 12.7 to 20.2%, smoking went from 16.1 to 17.4%, and total elevated cholesterol decreased from 38.1% in 1991–1994 to 32.9% in 2010–2012 (10). In studies of individual risk factors for NCDs in Mongolia, low physical activity increased by 16.3% from 2005 to 2013 (11), and the prevalence of hypertension among adults in China increased by 11.9% from 1991 to 2015 (12). In Myanmar (Yangon region), the prevalence of diabetes increased from 8.3% in 2004 to 10.2% in 2014 (13), and in Iran, the prevalence of daily smoking decreased from 31.1% in 1990 to 19.5% in 2016 among men and decreased from 5.4 to 1.0% among women in the same period (14).

Several sociodemographic factors, such as older age (5, 9, 15–17), being a male subject, educational level (9, 16), and urban residence (5, 16), have been found to increase the odds of multiple biological and behavioral risk factors for NCDs. The aim of the study was to assess trends (from 2007 to 2019) in NCD risk factors (low physical activity, insufficient fruit/vegetable intake, current tobacco use, problem alcohol use, diabetes, hypertension, elevated total cholesterol, and obesity) in Bhutan.

## Methods

Three cross-sectional Bhutan STEPS surveys (limited to the capital city of Thimphu in 2007, countrywide in 2014 and 2019) (18) with complete NCD risk factor measurements were analyzed; the overall response rate was >93% in 2014 > 96% in 2019 (19–21). Participants were randomly selected using a multi-stage stratified sampling procedure. One person per household within the age range of the survey (25–74 years in 2007, 18–69 years in 2014, and 15–69 years in 2019) was included (19–21). The study was approved by the Research Ethics Board for Health (REBH), Bhutan, and participants provided written informed consent.

Data collection followed the WHO 3-STEPS method: step 1—questionnaire administration (demographic, medical, and health risk behavioral information); step 2—blood pressure and anthropometric measurements; and step 3—biochemical tests (blood glucose and lipids) (18–21). Of the three blood pressure measurements, an Omron BP automatic blood pressure monitor apparatus was used (19–21); the last two readings were averaged (18). Blood glucose and elevated total cholesterol were measured in “peripheral (capillary) blood at the data collection site using dry chemical methods and biochemical analysis with a Hitachi 912 bio-analyzer” (19–21).

### Measures

#### Outcome variables

*NCD risk factors* were included based on previous studies (8, 15, 22, 23) as follows.

#### Behavioral NCD risk factors

Fruit/vegetable consumption (<5 servings/day), low physical activity according to the “Global Physical Activity Questionnaire” (24), current tobacco use, and problem alcohol use (eight or more standard drinks for women and 15 or more standard drinks for men/week) (11–21, 25).

#### Biological risk factors for NCDs

Diabetes was defined as “fasting plasma glucose levels ≥126 mg/dL, and/or currently taking insulin or oral hypoglycemic drugs” (18). Hypertension was assessed “based on measured blood pressure (BP; mean of the last two of three readings) defined as systolic BP ≥140 mm Hg and/or diastolic BP ≥90 mm Hg or currently on antihypertensive medication” (26); elevated total cholesterol (TC) was defined as plasma venous value ≥190 mg/dl; body mass index was measured as ≥25 kg/m^2^ obesity (27).

### Sociodemographics

Adult household members, residential and employment status, sex, age, and education (19–21).

### Data analysis

The study sample was “weighted considering the probability of selection at three levels and accounted for participant weight/ individual weight, non-response weight, and adjustment for participant’s age/sex group (population weight)” (19–21). The proportion of NCD risk factors was grouped based on previous studies (7, 15), 3–8 NCD risk factors (versus 0–2 risk factors), and the description of the NCD risk factors by study year is shown in bar graphs. Adjusted logistic regression was used to assess the predictors of each of the eight NCD risk factors, and linear regression was used to estimate the determinants of the number of NCD risk factors by study year. Only complete cases were included in the analysis. *p* < 0.05 was considered significant. Statistical procedures to account for the complex study design were performed using Stata SE 15.0 (College Station, TX, United States).

## Results

The sample consisted of 9,281 (≥15 years) participants (M = 41.7 years; SD = 13.7 years), 647 in 2005, 1,897 in 2009, 2,380 in 2013, and 5,004 in 2019. From 2007 to 2019, the proportion of older and urban residents decreased, and the proportion of those with higher education increased (Table 1).

**Table 1:** Sociodemographic characteristics of individuals aged 15 years and older in Bhutan, 2007, 2014, and 2019 (unweighted %).

Variable	Study year		
	2007	2014	2019
	*N* = 1897	*N* = 2,380	*N* = 5,004
	*N* (%)	*N* (%)	*N* (%)
Age (years)			
15–34	402 (21.2)	874 (36.7)	1,986 (39.7)
35–49	630 (33.2)	921 (38.7)	1,795 (34.7)
50–74	865 (45.6)	585 (24.6)	1,283 (25.6)
Sex			
Female subjects	1,042 (54.9)	1,466 (61.6)	3,095 (61.9)
Male subjects	855 (45.1)	914 (38.4)	1,909 (38.1)
Education (in years)			
0	1,303 (68.8)	1,455 (61.1)	2,492 (49.8)
1–10	386 (20.4)	656 (27.6)	1,529 (30.6)
≥11	206 (10.9)	269 (11.3)	983 (18.6)
Employment			
Non-employed	1,149 (61.2)	692 (29.2)	3,782 (75.6)
Employed	729 (38.8)	1,684 (70.8)	1,223 (24.4)
Adult household members			
0–2	573 (30.4)	1,642 (69.0)	1,416 (28.3)
3	500 (26.6)	427 (17.9)	1,230 (24.6)
4 or more	810 (43.0)	310 (13.0)	2,356 (47.1)
Residence			
Rural	0 (0.0)	747 (31.4)	1,881 (37.6)
Urban	1,897 (100)	1,633 (68.6)	3,123 (62.4)

### Distribution of NCD risk factors from 2007 to 2019

The prevalence of individual NCD risk factors increased significantly for inadequate fruit and vegetable intake from 64.3% in 2007 to 86.6% in 2019 (*p* < 0.001), for problem alcohol use from 3.1% in 2007 to 8.1% in 2019 (*p* < 0.001), and decreased significantly for low physical activity from 58.4% in 2007 to 11.4% in 2019 (*p* < 0.001), current tobacco use from 43.2% in 2007 to 23.4% in 2019 (*p* < 0.001), obesity (≥25 kg/m^2^) from 52.4% in 2007 to 43.1% in 2019 (*p* < 0.001), diabetes from 8.4% in 2007 to 2.6% in 2019 (*p* < 0.001), and total cholesterol from 42.2% in 2007 to 9.9% in 2019 (*p* < 0.001), while the prevalence of hypertension remained unchanged from 2007 to 2019. The proportion of people having 3–8 NCD risk factors decreased significantly from 62.8% in 2007 to 32.6% in 2019 (*p* < 0.001), and the mean number of NCD risk factors decreased significantly from 3.0 in 2007 to 2.1 in 2019 (*p* < 0.001). Similar results were found in the stratified analysis by sex (Table 2).

**Table 2:** Distribution of risk factors for non-communicable diseases among individuals aged 15 years and older in Bhutan, 2007, 2014, and 2019 (weighted %).

	Study year			*p* value
Non-communicable disease (NCD) risk factors	2007	2014	2019	
	*N* (%)	*N* (%)	*N* (%)	
All				
Fruit and vegetable intake (<5 servings/day)	1,262 (64.3)	1,619 (66.9)	4,317 (86.6)	<0.001
Low physical activity	1,178 (58.4)	325 (11.9)	597 (11.4)	<0.001
Current tobacco use	711 (43.2)	480 (24.8)	979 (23.4)	<0.001
Problem alcohol use	66 (3.1)	305 (12.8)	422 (8.1)	<0.001
General obesity (≥25 kg/m^2^)	1,029 (52.4)	903 (32.9)	2,562 (43.1)	<0.001
Hypertension	603 (26.0)	909 (35.7)	1,620 (27.0)	0.139
Diabetes	197 (8.4)	66 (2.3)	163 (2.6)	<0.001
Elevated total cholesterol	863 (44.2)	307 (12.5)	635 (9.9)	<0.001
3–8 NCD risk factors	1,275 (62.8)	780 (30.4)	1914 (32.6)	<0.001
	M (SD)	M (SD)	M (SD)	
Total NCD risk factors	3.0 (1.4)	2.0 (1.2)	2.1 (1.2)	<0.001
Male subjects				
Fruit/vegetable intake (<5 servings/day)	543 (62.1)	605 (66.0)	1,629 (86.6)	<0.001
Low physical activity	450 (50.8)	85 (8.2)	219 (10.9)	<0.001
Current tobacco use	324 (39.9)	286 (32.4)	587 (32.2)	<0.001
Problem alcohol use	37 (3.5)	166 (5.6)	239 (10.9)	<0.001
General overweight/obesity (≥25 kg/m^2^)	470 (52.4)	269 (26.7)	860 (38.9)	<0.001
Hypertension	27 (28.0)	353 (35.1)	73 (30.3)	0.108
Diabetes	89 (8.3)	25 (2.2)	62 (2.7)	<0.001
Elevated total cholesterol	382 (44.3)	120 (11.9)	203 (9.2)	<0.001
3–8 NCD risk factors	543 (59.8)	304 (30.8)	807 (35.3)	<0.001
	M (SD)	M (SD)	M (SD)	
Total NCD risk factors	2.9 (1.4)	2.0 (1.2)	2.2 (1.2)	<0.001
Female subjects				
Fruit/vegetable intake (<5 servings/day)	719 (67.3)	1,014 (70.4)	2,688 (86.8)	<0.001
Low physical activity	728 (69.8)	240 (16.8)	378 (12.3)	<0.001
Current tobacco use	387 (38.2)	194 (14.3)	392 (11.3)	<0.001
Problem alcohol use	29 (2.1)	139 (9.5)	183 (4.4)	<0.001
General overweight/obesity (≥25 kg/m^2^)	559 (53.6)	634 (39.8)	1702 (49.3)	<0.001
Hypertension	316 (22.9)	556 (35.2)	882 (23.1)	0.002
Diabetes	108 (8.5)	41 (2.3)	101 (2.6)	<0.001
Elevated total cholesterol	481 (41.1)	187 (11.9)	432 (11.0)	<0.001
3–8 NCD risk factors	732 (66.4)	476 (29.9)	1,107 (29.3)	<0.001
	M (SD)	M (SD)	M (SD)	
Total NCD risk factors	2.9 (1.4)	2.0 (1.2)	2.2 (1.2)	<0.001

**Figure 1:**
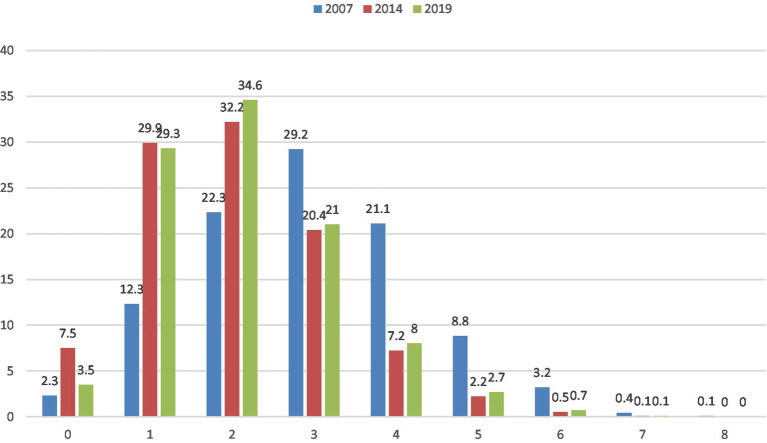
Prevalence of risk factors for non-communicable diseases among adults in Bhutan from 2007 to 2019 (%).

Across the study years, the prevalence of having zero NCD risk factors was 5.0%, 1 = 29.5%, 2 = 33.7%, 3 = 20.8%, 4 = 7.8%, 5 = 2.6%, 6 = 0.6%, and 7 = 0.1%, and eight risk factors were 0%; the distribution of the study year is shown in Figure 1.

### Sociodemographic determinants of individual behavioral NCD risk factors

Compared to the study year 2007, the study year 2019 had a significantly higher prevalence of inadequate fruit and vegetable intake (*p* < 0.001), and problem alcohol use (*p* < 0.001), and a significantly lower prevalence of low physical activity (*p* < 0.001) and current tobacco use (*p* < 0.001). The middle and/or older age groups had an increased probability of problem alcohol use (*p* < 0.001). Being a male subject decreased the odds of low physical activity (*p* < 0.001) and increased the odds of current tobacco use (*p* < 0.001) and problem alcohol use (*p* < 0.001). Higher levels of education were positively associated with low physical activity (*p* < 0.001) and inversely associated with problem alcohol use (*p* < 0.05). Being employed decreased the odds of inadequate fruit and vegetable intake (*p* < 0.001) and increased the odds of current tobacco use (*p* < 0.05) and problem alcohol use (*p* < 0.01; Table 3).

**Table 3:** Determinants of behavioral risk factors for non-communicable diseases.

Variable	Inadequate fruit/vegetable intake	Low physical activity	Current tobacco use	Problem alcohol use
	AOR (95% CI)	AOR (95% CI)	AOR (95% CI)	AOR (95% CI)
Study year				
2007	1 (Reference)	1 (Reference)	1 (Reference)	1 (Reference)
2014	1.37 (1.04, 1.82)^*^	0.10 (0.07, 0.12)^***^	0.37 (0.28, 0.48)^***^	4.41 (3.06, 6.37)^***^
2019	3.54 (2.63, 4.77)^***^	0.09 (0.06, 0.12)^***^	0.39 (0.30, 0.52)^***^	4.10 (2.96, 5.69)^***^
Age (years)				
15–34	1 (Reference)	1 (Reference)	1 (Reference)	1 (Reference)
35–49	0.88 (0.73, 1.06)	0.95 (0.75, 1.20)	0.87 (0.72, 1.06)	1.70 (1.29, 2.25)^***^
50–74	1.00 (0.79, 1.27)	1.22 (0.95, 1.55)	0.82 (0.65, 1.02)	1.90 (1.50, 2.50)^***^
Sex				
Female subjects	1 (Reference)	1 (Reference)	1 (Reference)	1 (Reference)
Male subjects	0.98 (0.82, 1.18)	0.60 (0.48, 0.70)^***^	3.31 (2.83, 3.89)^***^	2.06 (1.65, 2.55)^***^
Education (in years)				
0	1 (Reference)	1 (Reference)	1 (Reference)	1 (Reference)
1–10	0.91 (0.73, 1.13)	1.39 (1.13. 1.71)^**^	1.21 (0.99, 1.48)	0.74 (0.56, 0.99)^*^
≥11	0.82 (0.61, 1.11)	1.90 (1.31, 2.74)^***^	0.97 (0.74, 1.28)	0.64 (0.44, 0.92)^*^
Employment				
Non-employed	1 (Reference)	1 (Reference)	1 (Reference)	1 (Reference)
Employed	0.67 (0.56, 0.80)^***^	1.07 (0.84, 1.37)	1.31 (1.01, 1.69)^*^	1.52 (1.15, 2.01)^**^
Adult household members				
0–2	1 (Reference)	1 (Reference)	1 (Reference)	1 (Reference)
3	1.15 (0.91, 1.96)	0.77 (0.58, 1.02)	0.93 (0.75, 1.14)	0.76 (0.58, 0.99)^*^
4 or more	1.24 (0.94, 1.64)	0.96 (0.78, 1.19)	1.00 (0.80, 1.24)	0.84 (0.67, 1.07)
Residence				
Rural	1 (Reference)	1 (Reference)	1 (Reference)	1 (Reference)
Urban	1.13 (0.82, 1.57)	1.17 (0.83, 1.64)	1.12 (0.85, 1.47)	1.20 (0.92, 1.56)

^***^p < 0.001; ^**^p < 0.01; ^*^p < 0.05.

### Sociodemographic determinants of individual biological risk factors for NCDs

Compared to the study year 2007, the study year 2019 had a significantly higher prevalence of hypertension (*p* < 0.001) and lower prevalence of obesity (*p* < 0.01), lower diabetes (*p* < 0.001), and lower elevated total cholesterol (*p* < 0.001). Older age was associated with overweight/obesity (*p* < 0.001), hypertension (*p* < 0.001), diabetes (*p* < 0.001), and elevated total cholesterol (*p* < 0.001). Being a male subject was positively associated with hypertension (*p* < 0.05) and negatively associated with overweight/obesity (*p* < 0.001) and elevated total cholesterol (*p* < 0.05). Higher levels of education were associated with overweight/obesity (*p* < 0.05) and diabetes (*p* < 0.01) and being employed was associated with general obesity (*p* < 0.001) and hypertension (*p* < 0.001; Table 4).

**Table 4:** Determinants of biological risk factors for non-communicable diseases.

Variable	General obesity	Hypertension	Diabetes	Elevated total cholesterol
	AOR (95% CI)	AOR (95% CI)	AOR (95% CI)	AOR (95% CI)
Study year				
2007	1 (Reference)	1 (Reference)	1 (Reference)	1 (Reference)
2014	0.37 (0.30, 0.46)^***^	1.72 (1.42, 2.09)^***^	0.28 (0.19, 0.41)^***^	0.17 (0.14, 0.21)^***^
2019	0.77 (0.65, 0.90)^**^	1.48 (1.24, 1.76)^***^	0.39 (0.25, 0.62)^***^	0.14 (0.11, 0.17)^***^
Age (years)				
15–34	1 (Reference)	1 (Reference)	1 (Reference)	1 (Reference)
35–49	2.32 (1.98, 2.73)^***^	3.16 (2.69, 3.71)^***^	5.76 (2.85, 11.65)^***^	2.02 (1.51, 2.70)^***^
50–74	2.09 (1.72, 2.56)^***^	5.62 (4.51, 7.01)^***^	14.34 (6.71, 30.65)^***^	2.92 (2.13, 3.99)^***^
Sex				
Female subjects	1 (Reference)	1 (Reference)	1 (Reference)	1 (Reference)
Male subjects	0.54 (0.47, 0.61)^***^	1.18 (1.01, 1.38)^*^	0.73 (0.52, 1.04)	0.78 (0.63, 0.96)^*^
Education (in years)				
0	1 (Reference)	1 (Reference)	1 (Reference)	1 (Reference)
1–10	1.08 (0.93, 1.25)	0.91 (0.75, 1.10)	1.88 (1.23, 2.88)^**^	1.17 (0.94, 1.45)
≥11	1.25 (1.01, 1.54)^*^	0.85 (0.70, 1.03)	1.82 (0.92, 3.60)	0.93 (0.65, 1.34)
Employment				
Non-employed	1 (Reference)	1 (Reference)	1 (Reference)	1 (Reference)
Employed	1.52 (1.26, 1.83)^***^	1.37 (1.14, 1.64)^***^	1.19 (0.76, 1.87)	1.10 (0.86, 1.40)
Adult household members				
0–2	1 (Reference)	1 (Reference)	1 (Reference)	1 (Reference)
3	0.89 (0.75, 106)	0.97 (0.81, 1.16)	0.97 (0.69, 1.37)	1.02 (0.81, 1.21)
4 or more	0.86 (0.72, 1.02)	0.95 (0.79, 1.13)	0.82 (0.56, 1.19)	1.02 (0.80. 1.31)
Residence				
Rural	1 (Reference)	1 (Reference)	1 (Reference)	1 (Reference)
Urban	0.86 (0.73, 1.01)	0.93 (0.79, 1.10)	1.09 (0.61, 1.94)	0.83 (0.64, 1.07)

^***^p < 0.001; ^**^p < 0.01; ^*^p < 0.05.

### Associations with eight NCD risk factors

In linear regression analyses by study year, older age (*p* < 0.001) was positively associated with eight NCD risk factors. Furthermore, being a male subject (*p* < 0.01) was negatively and positively (*p* < 0.001) associated with eight NCD risk factors in 2007 and 2019, respectively. Higher levels of education (*p* < 0.05) were positively associated with eight NCD risk factors in 2007 and negatively associated (*p* < 0.05) with eight NCD risk factors in 2019. Employment (*p* < 0.001) and urban residence (*p* < 0.001) were positively associated with eight NCD risk factors in 2019, while urban residence (*p* < 0.001) was negatively associated with eight NCD risk factors in 2014 (Table 5).

**Table 5:** Associations with risk factors for non-communicable diseases among individuals aged 15 years and older in Bhutan, 2007–2019.

	Study year		
Variable	2007	2014	2019
	Coef. (95% CI)	Coef. (95% CI)	Coef. (95% CI)
All			
Age (years)			
15–34	Reference	Reference	Reference
35–49	0.36 (0.18 to 0.53)^***^	0.41 (0.27 to 0.55)^***^	0.57 (0.46 to 0.69)^***^
50–74	0.86 (0.67 to 1.04)^***^	0.78 (0.61 to 0.95)^***^	0.80 (0.65 to 0.94)^***^
Sex			
Female subjects	Reference	Reference	Reference
Male subjects	−0.24 (−0.39 to −0.09)^**^	−0.05 (−0.10 to 0.09)	0.16 (0.08 to 0.25)^***^
Education (in years)			
0	Reference	Reference	Reference
1–10	0.05 (−0.12 to 0.22)	0.15 (−0.02 to 0.31)	−0.12 (−0.20 to −0.03)^*^
≥11	0.26 (0.02 to 0.51)^*^	−0.04 (−0.29 to 0.21)	−0.18 (−0.31 to −0.04)^*^
Employment			
Non-employed	Reference	Reference	Reference
Employed	0.14 (−0.02 to 0.29)	0.04 (−0.11 to 0.19)	0.27 (0.16 to 0.38)^***^
Adult household members			
0–2	Reference	Reference	Reference
3	0.13 (−0.16 to 0.31)	−0.11 (−0.26 to 0.04)	−0.02 (−0.12 to 0.07)
4 or more	0.09 (−0.08 to 0.26)	0.01 (−0.18 to 0.21)	−0.01 (−0.12 to 0.10)
Residence			
Rural	---	Reference	Reference
Urban		−0.33 (−0.51 to −0.15)^***^	0.22 (0.09 to 0.36)^***^

^***^p < 0.001; ^**^p < 0.01; ^*^p < 0.05.

## Discussion

The study aimed to assess, for the first time, the trends (from 2007 to 2019) in NCD risk factors in Bhutan. We found that the prevalence of eight NCD risk factors decreased among Bhutanese adults from 2007 to 2019. Inadequate fruit and vegetable intake, problem alcohol use, and hypertension increased, and current tobacco use, low physical activity, obesity, diabetes, and elevated total cholesterol decreased from 2007 to 2019.

Consistent with studies in urban India (10) and China (12), the prevalence of hypertension increased over time in this study. The prevalence of diabetes increased over time in urban India (10) and Myanmar (13), but decreased in our study. Similar to a study in urban India (10), the prevalence of total elevated cholesterol decreased over time in our study. While a previous trend study in Mongolia (11) found an increase in low physical activity, our study found a decrease in low physical activity. Similar to a study in Iran (14), we observed a decrease in current tobacco use over time. In our study, the prevalence of obesity decreased over time, whereas it increased over time in urban India (10) and Mongolia (28).

In Bhutan, the decrease in current tobacco use may be attributed to tobacco demand reduction measures (total ban on tobacco sales, ban on tobacco advertising, and sponsorship) (29, 30), high compliance with health protection measures for non-smokers (29), and possibly changes in social norms (perception that tobacco use is a sin) (31). Decreases in low physical activity values may be attributed to the “implementation of community-wide public education and awareness campaigns for physical activity” (3, 29), the creation of open-air gyms, bicycle and hiking pathways, pedestrian days, and annual physical activity activities (32). Early diagnosis and better management of diabetes and elevated total cholesterol may have contributed to their reduction (3).

However, we found an increase in the prevalence of problem drinking despite having several measures in place to reduce harmful alcohol use (restrictions on physical availability, bans on alcohol advertising except in containers, and taxes on alcoholic beverages except spirits) (29). However, better enforcement of alcohol policies is needed in addition to public health promotion (33). Furthermore, since the main source of alcohol is home-brewed *Ara*, “intervention targeting a reduction in the production of the local alcohol *Ara* should be undertaken urgently” [*SIC*] (34). We also found an increase in the prevalence of inadequate fruit and vegetable consumption, which may be due to seasonal availability, dietary habits (8), and the decline in fruit production (35). The government can increase interventions in terms of body weight control, tobacco use cessation, reduction of alcohol use, healthy eating, and screening and control of high levels of blood glucose, cholesterol, and blood pressure.

In comparison to other low- and middle-income countries, the proportion of 3–8 NCD risk factors (62.8% in 2007 and 32.6% in 2019) in this study was higher than in Nepal (3–8 NCD risk factors, 27.7%) (9), Malawi (3–7 NCD risk factors, 16.5%) (22), and Uganda (3–5 NCD risk factors, 17.3%) (23). A high proportion of multiple NCD risk factors were found, as in a previous study in Bhutan (7), which increases the odds of developing NCDs in this country.

In line with previous research (7, 9, 15–17), this study shows that increasing age, being a male subject, being employed and living in an urban area in 2019, and having higher education in 2007 were associated with higher odds of having multiple NCD risk factors. Early screening targeting men, urban dwellers, and those with higher education should be promoted to prevent risk factors for NCDs in Bhutan. Compared to the 2013 Nepal paper, this study showed a higher rate of current tobacco use (24.8% in 2014) than in Nepal (18.5%), low physical activity (11.9% in 2014) than Nepal (3.4%), higher obesity (≥25 kg/m^2^, 32.9% in 2014) than Nepal (≥25 kg/m^2^, 21.4%), elevated blood pressure (35.7% in 2014) than Nepal (25.7%), and a lower rate of inadequate fruit and vegetable intake (66.9% in 2014) than Nepal (98.9%) (8), lower elevated blood glucose (2.3% in 2014) than Nepal (3.6%), and lower elevated total cholesterol (12.5% in 2014) than Nepal (22.7%) (9).

Current tobacco use, problem alcohol use, and hypertension were significantly higher in men than in women, while general obesity, low physical activity, and raised total cholesterol were significantly higher in women than in men. The higher prevalence of substance use in men than in women and the higher rate of overweight/obesity in women than in men have been observed in previous studies (15, 22). Higher levels of education were positively associated with low physical activity, obesity, and diabetes and inversely associated with problem alcohol use. Employment decreased the odds of inadequate fruit and vegetable intake and increased the odds of current tobacco use, problem alcohol use, general obesity, and hypertension. These results show how the eight specific NCD risk factors can be targeted differently according to sex, educational level, and employment status.

Study limitations include the cross-sectional, repeated survey design, and self-reporting of some indicators. The Bhutan 2007 STEPS survey was limited because it was subnational. The household income variable was collected in the three surveys, but there were missing cases, so this variable was excluded from the analysis.

## Conclusion

Based on three surveys of people aged 15 years or older, we found that the prevalence of eight NCD risk factors decreased in Bhutan from 2007 to 2019. Inadequate fruit and vegetable intake, problem alcohol use, and hypertension increased, and current tobacco use, low physical activity, obesity, diabetes, and elevated total cholesterol decreased from 2007 to 2019. Several factors associated with NCD risk factors were identified, including older age, sex, education level, and residence status, which may guide interventions. Future research may include a comparison of four surveys once the fourth Bhutan STEPS survey becomes available.

## Data availability statement

Publicly available datasets were analyzed in this study. This data can be found here: World Health Organization NCD Microdata Repository: Available at: https://extranet.who.int/ncdsmicrodata/index.php/catalog.

## Ethics statement

The studies involving human participants were reviewed and approved by the “Research Ethics Board for Health (REBH).” The patients/participants provided their written informed consent to participate in this study.

## Author contributions

SP and KP meet the criteria for authorship, conceived and designed the study, performed the statistical analysis, drafted the manuscript, and made critical revisions to key intellectual content. All authors contributed to the article and approved the submitted version.

## Conflict of interest

The authors declare that the research was conducted in the absence of any commercial or financial relationships that could be construed as a potential conflict of interest.

## Publisher’s note

All claims expressed in this article are solely those of the authors and do not necessarily represent those of their affiliated organizations, or those of the publisher, the editors and the reviewers. Any product that may be evaluated in this article, or claim that may be made by its manufacturer, is not guaranteed or endorsed by the publisher.
